# Mycoplasma pneumoniae among Hospitalized Patients with Acute Respiratory Tract Infections in an Indian Tertiary Care Hospital: an Underreported Health Problem

**DOI:** 10.1128/spectrum.01589-22

**Published:** 2022-07-14

**Authors:** K. Sreenath, S. K. Kabra, A. B. Dey, Amita Chandolia, Tanu Sagar, Vishwajeet Singh, Jagat Jeevan Ghimire, Randeep Guleria, Rama Chaudhry

**Affiliations:** a Department of Microbiology, All India Institute of Medical Sciences, New Delhi, India; b Department of Pediatrics, All India Institute of Medical Sciences, New Delhi, India; c Department of Geriatric Medicine, All India Institute of Medical Sciences, New Delhi, India; d Department of Biostatistics, All India Institute of Medical Sciences, New Delhi, India; e Department of Pulmonary, Critical Care, and Sleep Medicine, All India Institute of Medical Sciences, New Delhi, India; University of Mississippi Medical Center

**Keywords:** atypical pneumonia, CARDS toxin, *Mycoplasma pneumoniae*, molecular epidemiology, pneumonia, public health

## Abstract

The epidemiology of Mycoplasma pneumoniae (Mp) is poorly understood in India. The present study was conducted to identify the prevalence of Mp in a large set of patients with acute respiratory tract infections (ARI) in an Indian tertiary hospital. During 2015–2020, we tested throat swab specimens from patients with the clinical diagnosis of ARI (*n* = 1,098) by a real-time PCR and compared the demographic, clinical, laboratory, and outcome data of Mp-positive and Mp-negative patients. During the study period, 5% (55/1,098) of the tested samples were positive for Mp by PCR. School-aged children and young adults represented 36% (20/55) of the cases and 47.3% (26/55) of the cases were registered during the summer and monsoon. Among the Mp-positive patients, 61.8% (34/55) had underlying conditions; the most common were malignancy (*n* = 12; 21.8%) and hypertension (*n* = 6; 10.9%). Fever (98.2% versus 84.9%; *P* = 0.006), and pharyngitis (27.3% versus 16.3%; *P* = 0.034) were significantly common in the Mp-positive group than Mp-negative group. Among the Mp-positive group, 20% (11/55) of patients were admitted to an intensive care unit and a total of 7/55 (12.7%) patients received ventilatory support. The mortality in the Mp-positive cohort was 13.3%. The study provides baseline data regarding Mp prevalence and clinical characteristics. The application of molecular assays for diagnosing this pathogen among hospitalized patients with ARI could reduce inappropriate empirical antibiotic treatment and improve patient outcomes. Further large-scale studies are required to avoid the underdiagnosis of Mp infections in India and such studies should address some research gaps, such as macrolide resistance and molecular typing.

**IMPORTANCE** M. pneumoniae (Mp) is a significant pathogen causing atypical pneumonia but by far these infections are underreported clinical entities in India. In the present study, we report the prevalence of Mp and describe the demographic and baseline clinical data of Mp-positive cases in an Indian tertiary care hospital. Our study may improve the clinician’s awareness of this important agent of respiratory infection therefore timely and accurate diagnostic tools can be applied for patient management decisions and outcomes.

## INTRODUCTION

Mycoplasma pneumoniae (Mp) is an atypical bacterial pathogen that is implicated in acute respiratory infections (ARI) including, pharyngitis, tracheobronchitis, exacerbation of wheezing, and pneumonia (1). Infections can occur in the upper and lower respiratory tracts and a proportion of cases can lead to extrapulmonary manifestations including neurological, hematologic, gastrointestinal, and dermatological complications (2). Mp is one of the leading causes of community-acquired pneumonia (CAP) responsible for 20–40% of the reported cases (3). The clinical presentations may be benign or subclinical; however, respiratory manifestations can also include cellular bronchiolitis, bronchiolitis obliterans with organizing pneumonia or without organizing pneumonia, bronchiectasis, adult respiratory distress syndrome, pleural effusion, pulmonary embolism, and lung abscess (4–6). Fulminant and fatal pneumonia attributed to Mp can lead to respiratory failure and death (7).

The epidemiology of Mp among the Indian population with ARI is poorly understood. This is mainly due to the low availability of laboratory assays and physicians usually may not prescribe diagnostic tests for Mp. Diagnosis based on respiratory cultures is time-consuming and serology requires a convalescent-phase sample, limiting the clinical usefulness of these assays (8). PCR amplification from the respiratory secretions is reported to be more sensitive, specific, and time-effective for the detection and diagnosis of Mp pneumonia (4, 9, 10). As Mp is intrinsically resistant to many antibiotic classes typically used for the empirical broad-spectrum treatment of pneumonia, rapid identification of this pathogen has immediate clinical implications (11). The present study was conducted to identify the prevalence of Mp in patients with ARI in an Indian tertiary care hospital and describe the etiological, clinical, laboratory, radiographic, and outcome data of Mp-positive patients.

## RESULTS

### Study population.

During the study period, a total of 1,098 patients meeting the definition of ARI were enrolled and throat swab samples were tested for Mp. Among the enrolled patients, 616 (56.1%) were children <15 years old. The median age was 12 years (IQR = 1 month to 91 years), and the sex ratio (M/F) was 1.7. Of these (*n* = 1,098), 637 (58%) had at least one co-morbid conditions. Radiographic findings were available for 1079 (98.3%) patients and 897 (83.1%) had pneumonic changes on chest radiography. Of the 1098 patients, 365 (33.2%) were in an intensive care unit (ICU) and 190 (17.8%) patients received ventilatory support. A total of 262 (24.7%) patients in the ARI cohort died and 798 (75.3%) patients were discharged. Regarding antibiotic use, of the 771 (70.2%) patients of whom treatment information was available, most patients (*n* = 401 [52.1%]) were empirically treated with broad-spectrum antibiotics. Table 1 lists the baseline demographic and clinical features of all tested patients (*n* = 1,098).

**TABLE 1:** Demographic, clinical, and laboratory characteristics of patients with (Mp-positive) and without (Mp-negative) Mp infections^*a*^

Variable	Total (*n* = 1,098)	Available observations (*n*)	Mp-positive (*n* = 55)	Mp-negative (*n* = 1043)	*P*-value
Demographics, *n* (%)					
Male, sex	702 (63.93)	1,098	38 (69.09)	664 (63.66)	0.414
Adults	482 (43.9)	1,098	21 (38.18)	461 (44.2)	0.381
Children	616 (56.1)	1,098	34 (61.82)	582 (55.8)	
Age	12	1,098	9	13	0.149
Yr, median (min–max)	(1 mo–91)		(2 mo–81)	(1 mo–91)	
Age group, *n* (%)		1,098			
<1 yr	122 (11.11)		9 (16.36)	113 (10.83)	0.698
1–4 yr	194 (17.67)		8 (14.55)	186 (17.83)	
5–24 yr	358 (32.6)		20 (36.36)	338 (32.41)	
25–44 yr	141(12.84)		6 (10.91)	135 (12.94)	
45–65 yr	173 (15.76)		6 (10.91)	167 (16.01)	
>65 yr	110 (10.02)		6 (10.91)	104 (9.97)	
Seasonality, *n* (%)					
Winter	226 (20.58)	1,098	13 (23.64)	213 (20.42)	0.548
Spring	218 (19.85)		8 (14.55)	210 (20.13)	
Summer	294 (26.78)		19 (34.55)	275 (26.37)	
Monsoon	186 (16.94)		7 (12.73)	179 (17.16)	
Autumn	174 (15.85)		8 (14.55)	166 (15.92)	
Comorbidity, *n* (%)					
Any underlying medical conditions	637 (58.01)	1,098	34 (61.82)	603 (57.81)	0.558
Diabetes	100 (9.11)	1,098	5 (9.09)	95 (9.11)	0.997
Malignancy	181 (16.48)	1,098	12 (21.82)	169 (16.2)	0.274
Hypertension	116 (10.56)	1,098	6 (10.91)	110 (10.55)	0.932
Heart diseases	90 (8.2)	1,098	3 (5.45)	87 (8.34)	0.447
Neurological disorder	23 (2.09)	1,098	2 (3.64)	21 (2.01)	0.413
Renal diseases	14 (1.28)	1,098	2 (3.64)	12 (1.15)	0.109
Bronchial asthma	32 (2.91)	1,098	3 (5.45)	29 (2.78)	0.251
COPD	45 (4.1)	1,098	1 (1.82)	44 (4.22)	0.381
ARDS	15 (1.37)	1,098	2 (3.64)	13 (1.25)	0.137
Symptoms at presentation, *n* (%)					
Fever	939 (85.52)	1,098	54 (98.18)	885 (84.85)	**0.006** ^*b*^
Cough	858 (78.14)	1,098	47 (85.45)	811(77.76)	0.178
Pharyngitis	185 (16.85)	1,098	15 (27.27)	170 (16.30)	0.034
Hemoptysis	69 (6.28)	1,098	1 (1.82)	68 (6.52)	0.161
Dyspnea	715 (65.12)	1,098	29 (52.73)	686 (65.77)	**0.059**
Chest pain	129 (11.75)	1,098	7 (12.73)	122 (11.70)	0.817
Confusion	185 (16.85)	1,098	8 (14.55)	177 (16.97)	0.640
Relative bradycardia	33 (3.01)	1,098	1 (1.82)	32 (3.07)	0.597
Headache	75 (6.83)	1,098	4 (7.27)	71 (6.81)	0.894
Myalgia	166 (15.12)	1,098	8 (14.55)	158 (15.15)	0.903
Abdominal pain	139 (12.66)	1,098	6 (10.91)	133 (12.75)	0.689
Diarrhea	158 (14.39)	1,098	8 (14.55)	150 (14.38)	0.973
Pneumonic changes on chest radiography					
Infiltrations	502 (46.52)		18/53 (33.96)	484/1,026 (47.17)	0.325
Consolidation	190 (17.61)	1,079	12/53 (22.64)	178/1,026 (17.35)	
Pleural effusions	34 (3.15)	1,079	1/53 (1.89)	33/1026 (3.22)	
Ground glass opacity	32 (2.97)	1,079	2/53(3.77)	30/1026 (2.92)	
Reticular or linear opacities	90 (8.34)	1,079	4/53 (7.55)	86/1026 (8.35)	
Cavitation	5 (0.46)	1,079		5 (0.49)	
Lung collapse	16 (1.48)	1,079		16 (1.56)	
Bronchial wall thickening	6 (0.56)	1,079		6 (0.58)	
Others	22 (2)	1,079		22 (2.14)	
Laboratory findings					
Baseline blood test, mean (min–max)					
White blood cell count (x10^3^ cells/μL)	10.27 (0.12–45)	1,095	9.5 (0.88–35.24)	10.3 (0.12–45)	0.810
Absolute neutrophil counts	69 (2–101)	1,074	59 (16–96)	70 (2–101)	**0.002**
Absolute lymphocyte count	22 (1–100)	1,065	30 (2–83)	22 (1–100)	0.954
Platelet count (x10^3^ cells/μL)	171 (5–600)	1,078	163 (20-480)	171 (5–600)	0.295
C-reactive protein (mg/dL)	24 (0.02–883)	458	17 (0.02–585)	25 (0.05–883)	0.970
Abnormal WBC count, *n* (%)^*c*^					
Leukopenia	158 (14.43)	1,095	6 (10.91)	152/1,040 (14.62)	0.446
Leukocytosis	484 (44.2)	1,095	23 (41.82)	461/1,040 (44.33)	0.715
Abnormal platelet count, *n* (%)^*d*^					
Thrombocytopenia	457 (42.39)	1,078	22/52 (42.31)	435/1,026 (42.4)	0.990
Thrombocytosis	107 (9.93)	1,078	5/52 (9.62)	102/1,016 (9.94)	0.939
Elevated AST^*e*^	251 (23.5)	1,068	8/50 (16)	243/1,018 (23.87)	0.2
Elevated ALT^*f*^	252 (23.57)	1,069	14/50 (28)	238/1,019 (23.36)	0.450
Elevated CRP (>6 mg/dL)	326 (71.18)	458	13/17 (76.47)	313/441 (70.98)	0.623
Hyponatremia^*g*^	276 (25.53)	1,081	9/51 (17.65)	267/1,030 (25.92)	0.186
Severity of illness, n (%)					
Admission to an ICU	365 (33.24)	1,098	11 (20.00)	354 (33.94)	**0.032**
Required ventilatory support	190 (17.82)	1,066	7 (12.73)	183/1,011 (18.10)	0.311
Hospital stays duration, days median (min-max)	12 (1–269)	1,089	10 (1–36)	12 (1–269)	**0.010**
Outcome					0.70
Died	262 (24.72)	1,060	6/44 (13.33)	256/1,015 (25.22)	
Survived	798 (75.28)	1,060	39/44 (86.67)	759/1,015 (74.78)	
Treatment, *n* (%)					
Any inpatient antibiotic					
Penicillin’s/cephalosporins	207 (26.85)	771	9/44 (20.45)	198/727 (27.24)	0.324
Macrolide	233 (30.22)	771	15/44 (34.09)	218/727 (29.99)	0.565
Quinolones	137 (17.77)	771	9/44 (20.45)	128/727 (17.61)	0.631
Tetracyclines	38 (4.93)	771		38/727 (5.23)	0.100
Combination antibiotics	401 (52.01)	771	22/44 (50)	379/727 (52.13)	0.783

a ALT, alanine aminotransferase; ARDS, acute respiratory distress syndrome; AST, aspartate aminotransferase; COPD, chronic obstructive pulmonary disease; CRP, C-reactive protein; ICU, intensive care unit; WBC, white blood cells.

b Bold text indicates statistical significance. *P*-value denotes the comparison between Mp-positive and Mp-negative groups.

c Reference range is 4–11 × 10^3^ cells/μL; WBC counts <4,000 or >11,000 cells/μL were considered abnormal.

d Reference range is 150–400 × 10^3^ cells/μL; platelet counts <150,000 or >400,000 cells/μL were considered abnormal.

e Reference range is 5–40 U/L.

f Reference range is 5–42 U/L.

g Reference range 135–145 mmol/L; serum sodium level <135 mmol/L was considered as hyponatremia.

### Mp detection rate and epidemiology.

Of the 1,098 patients with ARI, Mp was detected in 55 (5%) patients. The median age of Mp-positive patients was 9 years (IQR = 2 months to 81 years); 34 (61.82%) were children, and 38 (69.1%) were males. There was no significant difference in sex and age between the patients with ARI with Mp-positive and negative groups (*P* > 0.05). Among the 55 patients with Mp-positive results, 9 (16.4%) patients were aged <1 year, 8 (14.5%) were 1–4 years, 20 (36.4%) were aged 5–24 years, and 6 (10.9%) each patient aged 25–44 years, 45–65 years, and >65 years, respectively. In this study, Mp was most prevalent among the school-aged children and young adults (*n* = 20; 36.4%) even though the difference was not statistically significant (*P* > 0.05).

**FIG 1:**
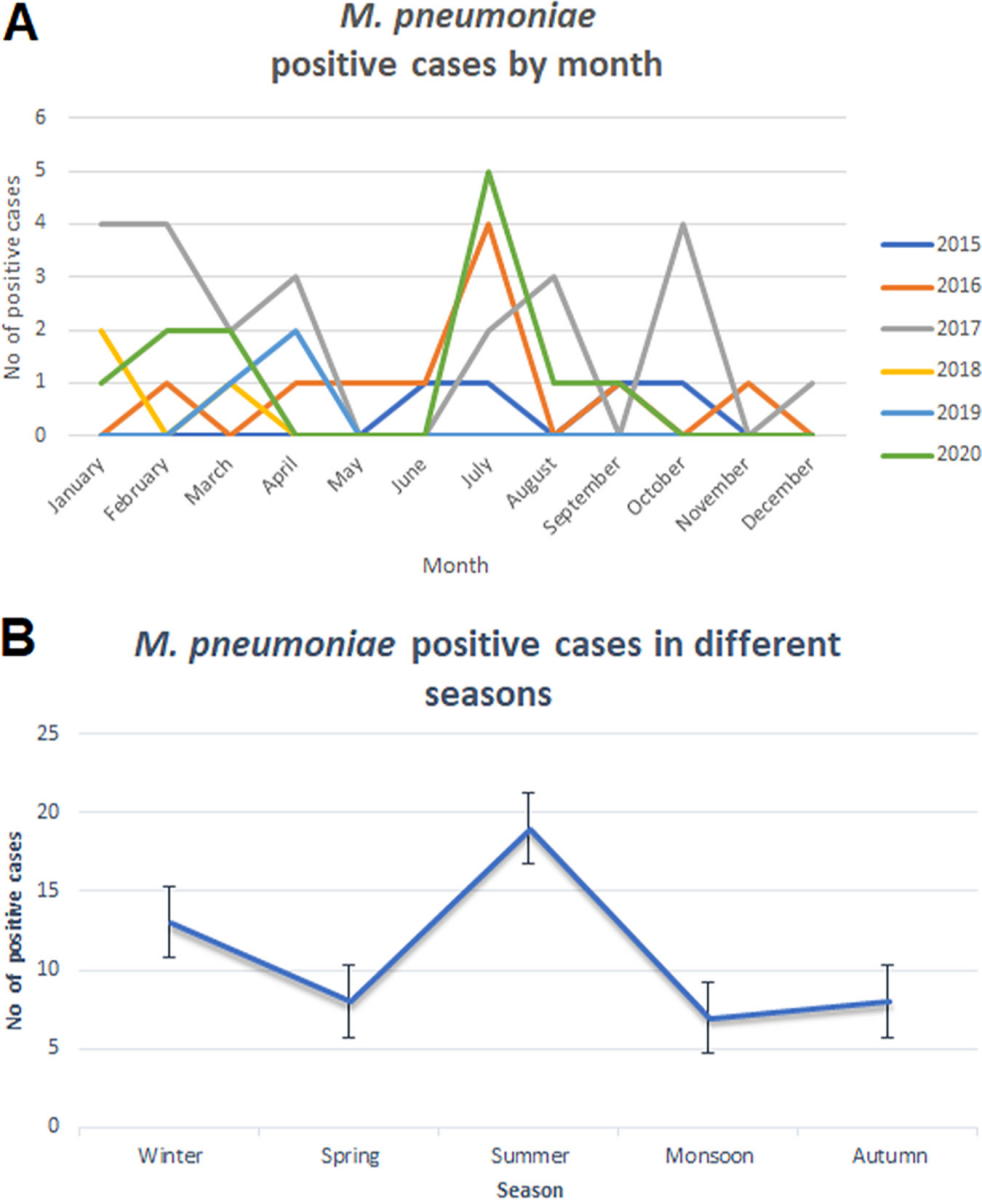
(A) No. of M. pneumoniae*-*positiv*e* cases (*n* = 55) were stratified by month during a 5-year study period. (B) Detection of M. pneumoniae (no. of cases) in different seasons during a 5-year study period. The study site (Delhi) has five distinct seasons, *viz.* Winter; December-January, Spring; February-March, Summer; April-June, Monsoon; July-September, Autumn; October-November.

The monthly distribution of Mp infection at our study site is shown in Fig. 1. At our study site, most of the Mp-positive cases were reported during July month (*n* = 12; 21.8%). The prevalence of Mp was higher during the summer and monsoon (April to September, *n* = 26; 47.27%; Fig. 1) but without statistical significance (*P* > 0.05).

### Clinical characteristics.

The demographic and clinical characteristics of Mp-positive patients are shown in Table 1. Systemic underlying diseases were observed in 34 (61.8%) patients, such as malignancy (*n* = 12; 21.8%), hypertension (*n* = 6; 10.9%), diabetes mellitus (*n* = 5; 9.1%), heart disease (*n* = 3; 5.5%), bronchial asthma (*n* = 3; 5.5%), renal disease (*n* = 2; 3.6%), and neurological disorders (*n* = 2; 3.6%). There were no significant differences in the underlying medical conditions between ARI patients with confirmed Mp infection and those without Mp infection (*P* > 0.05) (Table 1).

The most common symptoms in Mp-positive patients include fever (*n* = 54; 98.2%), cough (*n* = 47; 85.5%), dyspnea (*n* = 29; 52.7%), pharyngitis (*n* = 15; 27.3%), confusion (*n* = 8; 14.5%), myalgia (*n* = 8; 14.5%), diarrhea (*n* = 8; 14.5%), chest pain (*n* = 7; 12.7%), and abdominal pain (*n* = 6; 10.9%). Of these, only fever (98.2% versus 84.9%; *P* = 0.006) and pharyngitis (27.3% versus 16.3%; *P* = 0.034) were significantly more common in Mp-positive group compared to Mp-negative group (Table 1). Pneumonic changes in the chest radiography were reported in 37/53 (69.81%) patients; infiltrations (*n* = 18) were the most common pattern followed by consolidations (*n* = 12), reticular opacities (*n* = 4), ground glass opacities (GGO, *n* = 2), and pleural effusion (*n* = 1). These patterns were not significantly different between the Mp-positive and negative groups (*P* > 0.05). Extrapulmonary manifestations were also comparable in the Mp-positive and negative groups (40% versus 37%, *P* > 0.05) (Table 1).

### Laboratory parameters.

Among the Mp-positive patients, the most frequent hematological findings included leukocytosis (41.8%; *n* = 23/55), and thrombocytopenia (42.3%; *n* = 22/52) although they were not statistically significant (*P* > 0.05). No significant differences in the proportions of laboratory characteristics between ARI patients with confirmed Mp and those without Mp were found, except for neutrophil counts. The absolute neutrophil count for Mp-positive patients was significantly lower than that of the Mp-negative group (56.85 ± 22.42 versus 65.12 ± 21.16, *P* = 0.002) (Table 1). Besides, Mp- positive patients were less likely to have lymphopenia (28.3% versus 42.3%; *P* = 0.044) compared to the Mp-negative group. Of the Mp-positive cases, the detection of anti-Mp IgM antibodies (Nova Tec Immundiagnostica, Germany) were performed in 16 (29.1%) and 9 (56.3%) were positive. Of the Mp-positive patients, all (*n* = 55) were negative for Legionella pneumophila by *mip* gene PCR and *Legionella* urinary antigen test (BinaxNOW, Alere). In total, three (5.5%) Mp-positive patients had a positive result for SARS-CoV-2 by real-time reverse transcription-PCR. Microbiological results regarding the other bacterial and viral pathogens were not available.

### Treatment and prognosis.

Among the Mp-positive cases, information regarding antibiotic treatment was available for only 44 (80%) patients. Of these (*n* = 44), 23 (52.3%) received anti-Mp treatment; 15 (34.1%) received a macrolide (azithromycin) antibiotic; and 9 (20.5%) received fluoroquinolones (levofloxacin; *n* = 6, ciprofloxacin, *n* = 3). One patient received a combination treatment of both azithromycin and levofloxacin. Among the Mp-positive cohort, 11 (20%) patients were admitted to an ICU and a total of 7 (12.7%) patients received ventilatory support. Mp-positive patients were less likely to require ICU admissions (20% versus 33.9%, *P* = 0.032) than the Mp-negative patients (Table 1). Both Mp-positive adults and children (61.90% versus 38.10%; 91.18% versus 8.82%, *P* = 0.008) were more likely to be admitted to the hospital wards than the ICU. The proportion of Mp-positive patients with dyspnea in the general wards was significantly higher than those in the ICU (65.52% versus 34.48%, *P* = 0.05). The proportion of Mp-positive patients with abdominal pain was significantly higher in the ICU compared to general wards (66.67% versus 33.33%, *P* = 0.002). Mp-positive patients in the ICU were more likely to require ventilatory support than those in general wards (85.71% versus 14.29%, *P* =< 0.001). Other demographic and clinical characteristics did not show a significant difference between Mp-positive patients in the general ward and ICUs. (Table 2). Mp-positive patients had a shorter hospital length of stay compared to the Mp-negative group (11 ± 8 days versus 18 ± 20 days, *P* = 0.01). Of the Mp-positive patients, the outcome information was available for 45(81.8%) and 6 (13.3%) patients were expired. Of these, 5 (83.3%) patients had at least one co-morbid condition including heart disease, diabetes mellites, renal disease, neurological condition, and ARDS (a case of co-infection with Mp and SARS-CoV-2).

**TABLE 2:** Comparison of demographic and clinical features of Mp-positive patients (*n* = 55) in ICU and general wards

Variable	ICU patients *n* (%)	General ward patients *n* (%)	*P*-value^*a*^
Demographics			
Adult	8 (38.10)	13 (61.90)	**0.008**
Children	3 (8.82)	31 (91.18)	
Male	6 (5.79)	32 (84.21)	0.243
Female	5 (29.41)	12 (70.59)	
Patients with co-morbid conditions	6 (17.65)	28 (82.35)	0.579
Clinical presentations			
Fever	11 (20.37)	43 (79.63)	0.614
Chills	4 (36.36)	7 (63.64)	0.129
Pharyngitis	4 (26.64)	11 (73.33)	0.449
Cough	10 (21.28)	37 (78.72)	0.566
Dyspnea	10 (34.48)	19 (65.52)	**0.005**
Chest pain	2 (28.57)	5 (71.43)	0.544
Confusion	3 (37.5)	5 (62.5)	0.181
Headache	1 (25)	3 (75)	0.795
Myalgia	3 (37.5)	5 (62.5)	0.181
Abdominal pain	4 (66.67)	2 (33.33)	**0.002**
Diarrhea	3 (37.5)	5 (62.5)	0.181
Pneumonic changes in chest radiography	8 (21.62)	29 (78.38)	0.813
Complications			
Requirement of ventilatory support	6 (85.71)	1 (14.29)	**<0.001**
Duration of hospital stay, days median (min-max)	10 (5-33)	9.5 (1-36)	0.240
Outcome			
Survived	8 (20.51)	31 (79.49)	
Expired	2 (33.33)	4 (66.67)	0.482

a Bold text indicates statistical significance. *P*-value denotes the comparison between cases in the ICU and general ward groups.

## DISCUSSION

### Principal findings.

The present study was conducted to determine the prevalence of Mp and to evaluate the clinical profile of patients admitted for ARI due to Mp. PCR was reported to be the current gold standard for the detection of Mp because of its superior sensitivity and specificity compared to serology; however, the persistence of Mp in the respiratory tract even after antimicrobial treatment needs to be considered (4, 9, 10, 12). Baseman et al. reported that the CARDS toxin gene sequences are more sensitive for the identification of Mp than other gene targets, such as the P1 adhesin molecule and the *ATPase* gene (13). In the present study, using a CARDS toxin PCR assay, we identified Mp in 5% of patients’ biospecimens during the 5-year study period.

In our study, Mp infections were more common in school-aged children and young adults, and most cases were reported during the summer and monsoon. Similar findings have been presented in other studies (4, 14). In literature, it is reported that Mp epidemics occur every 3 to 7 years, and even though cases occur throughout the year, the peak is usually at the end of summer to early winter (15). In our cohort, patients with Mp infections had significantly lower ICU admission rates and duration of hospital stay which agrees with a previous study from Taiwan (16). However, long febrile periods, prolonged clinical course, and longer hospital stay due to Mp pneumonia (MPP) has been reported in previous studies (17, 18).

### Literature comparison.

The positive rate of Mp differs depending on the study period and population, age group, identification method, and type of bio samples. In France, detection rates of Mp varied from 2% to 10% during 5 years among outpatients with an ARI (19). During 2010–2012, in a U.S. multicentric study, 8% of hospitalized children with CAP were PCR-positive for Mp as reported by Kutty et al. (20). Carrim M et al. reported Mp prevalence of 1.6% and 0.7% among patients with severe respiratory illness and influenza-like illness, respectively in Africa from 2012 to 2015 (21). During 2011-to 2014, a former study from our group reported an overall Mp detection rate of 11.4%, 3.8%, and 1.5% by IgM assay, culture, and *P1* gene PCR, respectively (22). The Mp detection rates reported in this present study fall within these ranges. However, higher detection rates of 25% have been reported in Peruvian children hospitalized with ARI (23). In the United States and Finland, higher positivity rates of 27% to 30% have been reported among children with CAP (21, 24). Additionally, an Mp detection rate of up to 60% has been registered among hospitalized adults with pneumonia in Japan (25). The difference in detection rates in this study might be attributable to a difference in the patients enrolled, their age group, patient co-morbidities, the diagnostic assay used, use of antibiotics before hospital admission, and temporal or geographical variation in Mp activity.

In our study, fever and pharyngitis were significantly more common in the Mp-positive group. The clinical features reported in the present study show resemblance to those reported by other investigators (21, 23, 26). Generally, the signs, symptoms, radiographic, and laboratory features may not be helpful to differentiate Mp from other bacterial and viral types of pneumonia. Therefore, clinicians usually rely on microbiological testing to detect Mp. On radiography, Mp-positive patients in our cohort showed infiltrations, consolidations, reticular and GGO, and pleural effusion; however, the differences in radiographic features between Mp-positive and negative groups were not significant. Literature shows the most frequent radiographic findings in MPP are unilateral or bilateral areas of air-space consolidation and GGO. These findings may vary and could include reticular and nodular opacities, bronchial wall thickening, and pleural effusion (27, 28).

In this study, only half of the Mp-positive patients received the antibiotic having atypical coverage including, macrolides and quinolones. This is because Mp PCR was retrospectively performed in a few patients and the results were not available for treatment decisions. We suggest rapid molecular testing for Mp allows for targeted treatment; therefore, improves patient outcomes, mitigates disease spread, limits hospital stay duration, and reduces unnecessary antibiotic prescriptions.

Among the Mp-positive group, three patients had co-infection with SARS-CoV-2. Co-infection with SARS-CoV-2 and other typical and atypical bacterial pathogens has been reported in previous studies (29–31). For one patient in our cohort, the co-infection with SARS-CoV-2 and Mp was fatal. The clinical course of this patient has been reported in a previous publication by our group (32). Patients with COVID-19 and MPP may have a similar clinical presentation and radiographic features; therefore, all critically ill patients with COVID-19 should be meticulously tested for other atypical pathogens including Mp to ensure adequate treatment.

### Strength and limitations.

The strength of our study is the application of a sensitive and specific assay in a large set of patients enrolled in the same center for 5 years. The study provides information regarding Mp prevalence for clinicians and baseline data for future Mp surveillance programs. Our study has several limitations. First, we enrolled hospitalized patients with ARI; however, the Mp prevalence reported in this study mainly represents lower respiratory tract ARI. Second, we could not perform Mp culture to isolate Mp from PCR-positive specimens. Also, the Mp PCR was retrospectively performed in a few patients; therefore, the results were not communicated to treating clinicians. Third, a few patients in our cohort have received antibiotics with Mp activity before collecting throat swab specimens. This might have affected Mp-positivity. Fourth, respiratory culture and PCR results of other pathogens for ARI patients in our cohort were not collected. Therefore, information regarding co-infections and other common etiologies causing ARI were not included in this report. Fifth, the treatment information for Mp-positive patients was limited. Finally, this is a single-center study; therefore, the study findings cannot be generalized and should be carefully extrapolated. Pathogens’ prevalence may vary in different regions with different demographic patterns, climates, and health care accessibility.

### Conclusion.

In conclusion, we found a low prevalence of Mp infection in ARI patients admitted to an Indian tertiary care hospital. Data also shed some light on the epidemiology, clinical, and laboratory features of Mp infections in this region. Mp prevalence was more common in school-aged children and young adults. Even though the course of ARI due to Mp is usually self-limiting, we found that 20% of people needed ICU admission and 13% died. Increasing access to molecular tests including real-time PCR could facilitate a definitive diagnosis of this pathogen. The timely identification of MPP could reduce complications including extrapulmonary manifestations and can guide the clinicians in the use of targeted therapy.

### Implications for future work.

Macrolide resistance in Mp has been identified as a public health problem in Europe and the United States and the reported rates of macrolide-resistant Mp (MRMP) are as high as 90% in Asia (33, 34). Literature suggests that macrolide-resistant Mp (MRMP) patients may have a prolonged course of fever and cough compared to macrolide-sensitive strains (35). In India, data is needed regarding the MRMP prevalence and its clinical implications to inform antibiotic prescribing decisions. Globally, two main genotypes of Mp have been identified so far (type 1 and 2) based on the differences in the sequence of the *P1 adhesin* gene, and the type 2 strains are reported to be more toxigenic than type 1 (36). Presently, there is no information regarding the circulating Mp genotypes in our country. Genotyping data from our region may be useful to monitor the disease trends over time, especially during Mp outbreak investigations. Finally, to understand the exact burden of Mp in the Indian population, larger multicentric studies are needed to register the epidemiology, clinical course, and prognostic factors of Mp infections and the carriage of this pathogen in the general population.

## MATERIALS AND METHODS

### Patients and samples.

Both adults and children presenting to the emergency department, general wards, or an intensive care unit (ICU) of the All India Institute of Medical Sciences (AIIMS), New Delhi, India with the evidence of ARI, were enrolled in this study from February 2015 to February 2021. The definition of ARI cases was as follows: a respiratory infection with an acute fever of ≥38°C, and either a cough or sore throat or difficulty breathing, onset within the last 10 days, requiring hospitalization (37). Patients presenting to the emergency department with ARI and transferred later to other wards were excluded to avoid duplication. The institutional review board of the AIIMS has approved this study (approval no IECPG-169/27.01.16) and all patients were enrolled after obtaining informed written consent. A throat swab in transport media (suspended in 2 mL of the pleuro-pneumonia-like organism [PPLO] broth medium [BD, Difco, Mumbai, India]) was obtained from each enrolled patient and transported to the laboratory for Mp testing.

### DNA extraction and real-time PCR for Mp CARDS toxin gene detection.

Swab samples were vortexed for around 1 min after arriving at the study site (Microbiology Laboratory, AIIMS, New Delhi) and the swabs were discarded. Genomic DNA was extracted from 200 μL of PPLO broth specimen using the QIAamp DNA minikit (Qiagen, Hilden, Germany) following the manufacturer’s instructions. DNA was eluted in a final volume of 100 μL and preserved at −20°C prior to testing. The presence or absence of Mp was determined by analysis with real-time PCR for CARDS toxin nucleotide sequences according to a previously published and validated method (38). Briefly, reactions were performed in a final volume of -25 μL, consisting of 12.5 μL of TaqMan Universal PCR Master Mix (Applied Biosystems, MA), 0.5 μM final concentration of each primer, and 0.1 μM final concentration of probe (Mp181-F-TTTGGTAGCTGGTTACGGGAAT, Mp181-R-GGTCGGCACGAATTTCATATAAG, Mp181-P- FAM-TGTACCAGAGCACCCCAGAAGGGCT-MGBNFQ, Applied Biosystems, Warrington, UK), 5 μL of template DNA, and nuclease-free water (Promega, Mumbai, India) to achieve the final volume. The assay was performed on a step-one plus real-time PCR system (Applied Biosystems, MA) under the following conditions: initial activation of 95°C for 5 min, followed by 45 cycles of 95°C for 10 s and 60°C for 30 s.

The analytical specificity of the real-time PCR was checked using the following panel of organisms: Mycoplasma hominis, Mycoplasma genitalium, Mycoplasma capricolum, L. pneumophila, Chlamydia pneumoniae, Escherichia coli, Haemophilus influenzae type b, Klebsiella pneumoniae, Mycobacterium tuberculosis, Pseudomonas aeruginosa, Staphylococcus aureus, Streptococcus agalactiae, Streptococcus pneumoniae, Streptococcus pyogenes, Staphylococcus epidermidis, Ureaplasma urealyticum, Candida albicans, and Neisseria meningitidis. Primer pairs did not react with any other organisms tested; therefore, no amplification was observed for these non-Mp-tested organisms. The analytical sensitivity of the PCR was assessed by testing a serial dilution of the CARDS toxin gene cloned in the pUC19 vector (10^6^,10^5^, 10^4^, 10^3^, 10^2^, and 10^1^ copies/μL) and the limit of detection (LOD) was found to be 10 copies/μL. Briefly, using CARDS toxin-specific primers MPN-372-FP-5′-TTC CAC TTC AGA AAC ACC CAC AGC-3′ and MPN-372-RP-5′-TCA ATC AGG GCA CGC AAA CG-3′, we amplified 556 bp segment of Mp CARDS toxin gene. The 556 bp PCR product from Mp M129 (ATCC, USA) standard strain was then cloned into a pUC19 vector according to the manufacturer’s instructions. The positive clone was confirmed by restriction digestion and sequencing. This plasmid was serially diluted and used for determining the LOD of Mp real-time PCR assay. The real-time PCR was performed as described in the experimental procedures and in each experiment, 5 μL of each concentration of dilution rows (from 10^6^ to 10^1^ copies/μL) in a 25-μL reaction was tested. The LOD was evaluated and found to be 10 copies/μL.

Results of real-time PCR for Mp on throat swabs were considered positive at a cycle threshold (Ct value) of ≤40. When a positive result was obtained, the specimen was re-extracted and tested in triplicates for conformation. A sample was determined PCR-positive for the pathogen if tested positive in at least two of the three repeats. Genomic DNA extracted from Mp M129 B7 (ATCC 29342) was used as a positive control for real-time PCR. An ARI patient with a positive Mp real-time PCR throat swab specimen was considered to have ARI due to Mp (Mp-positive). If Mp- was not detected by real-time PCR on a tested sample, the patient was considered to have ARI without Mp (Mp-negative).

### Clinical data collection.

Demographic and clinical data from each subject was collected using a standardized questionnaire or from electronic medical records. The details include patient demographics, baseline clinical data, laboratory findings, medical interventions including the antibiotic treatment, complications (such as the requirement of ventilatory support, admission to an ICU), and outcome. Whenever available, chest radiographs were obtained and evaluated by attending physicians. We considered the following antibiotics having anti-Mp activity: macrolides (e.g., azithromycin, clarithromycin, erythromycin), fluoroquinolones (e.g., ciprofloxacin, moxifloxacin, levofloxacin), and doxycycline.

### Statistical analysis.

Categorical variables were expressed as numbers (percentages) and compared between two groups (Mp-positive versus Mp-negative) using the chi-square or Fisher’s exact tests, as appropriate. Continuous variables were shown as median (ranges) and/or mean ± SD and compared between two groups using the *t* test or Wilcoxon rank-sum test according to the distribution of the data. All the statistical analyses were performed using the statistical software STATA 14.2. Statistical significance was defined at a *P* value of less than 0.05.

### Data availability.

All relevant data are available within the article. Further inquiries can be directed to the corresponding author.
